# Genomic-Wide Association Markers and Candidate Genes for the High-Protein Trait in Storage Roots of Cassava (*Manihot esculenta*)

**DOI:** 10.3390/plants14203162

**Published:** 2025-10-15

**Authors:** Dantong Wang, Qi Liu, Xianhai Xie, Junyu Zhang, Jin Xiao, Wenquan Wang

**Affiliations:** 1 Sanya Institute of Breeding and Multiplication, School of Tropical Agriculture and Forestry, Hainan University, Sanya 572025, China; 23220951310202@hainanu.edu.cn (D.W.);; 2 National School of Tropical Agriculture and Forestry, Hainan University, Sanya 571700, China

**Keywords:** cassava, protein content, single-nucleotide polymorphism (SNP), genome-wide association study (GWAS), candidate genes

## Abstract

Cassava (*Manihot esculenta* Crantz) is a globally important staple crop. Although its leaves are rich in crude protein, the protein content in its storage roots is typically less than 2%, which limits its nutritional value. Exploring high-protein storage root genotypes from germplasm collections is essential to elucidate the mechanisms underlying protein allocation, yet this remains poorly understood. Here, we conducted a three-year field evaluation of protein content in storage roots of 261 lines derived from a hybrid population (SC205*18R). It was found that there were 21 lines with high protein content that was stably above 4%. A total of 22 significant associated loci of protein content in storage roots were identified through genome-wide association analysis, with their contribution rates ranging from 0.12 to 0.35. For instance, the haplotypes of SNP-6831776 and SNP-7090537 have a prominent contribution to the protein content in the storage roots and can be used as major-effect markers in breeding. Based on this, we found 82 candidate genes, 7 of which exhibited the strongest and most consistent associations with root protein accumulation. qRT-PCR validation demonstrated that six candidate genes were significantly upregulated in high-protein varieties. These resources and findings provide a crucial foundation for breeding for storage roots with high protein and enhancing the nutritional and economic value of cassava.

## 1. Introduction

Cassava (Manihot esculenta Crantz), also known as manioc, is a tuberous crop belonging to the Euphorbiaceae family and ranks as the sixth most important food crop worldwide. It is widely distributed between 30° N and 30° S latitude and below 2000 m elevation, with a global cultivation area of 28 million hectares and an annual fresh-root yield of 290 million tons [1], second only to potato. Cassava provides staple food for more than 800 million people, especially in sub-Saharan Africa [2], where its exceptional drought tolerance makes it a critical food security crop. In Central and South America and Southeast Asia, cassava is also a daily dietary component and a major source of calories in tropical regions. In industrialized Asian countries, cassava serves as an important feedstock, energy source, and raw material for green chemical production [3]. In China, cassava is cultivated on over 400,000 ha, mainly in the tropical and subtropical provinces of Guangxi, Guangdong, and Hainan. Approximately 90% of domestic production is used for starch extraction, while imports exceed domestic output by more than fivefold, resulting in a self-sufficiency rate of only 20% [3].

Storage roots of cultivated cassava are starch-rich, with starch concentrations generally ranging from 25% to 34%, earning cassava the title “king of starch crops” [2]. However, storage root protein content is only 2%, markedly lower than that of major cereals such as wheat, maize, and soybean [4,5]. Nonetheless, protein distribution among different organs of cassava is highly heterogeneous; leaves contain 5–7 g protein per 100 g fresh weight and 22–24% on a dry-weight basis, presenting a clear nutritional advantage [6]. The amino-acid profile is well-balanced, except for relatively low methionine, and is rich in arginine, glutamate, aspartate, and lysine, indicating high nutritional value [7]. These proteins play key roles in plant growth, development, and stress tolerance, while also providing essential nutrients for human consumption. Therefore, increasing storage root protein content in cassava is of great significance for enhancing both its nutritional and economic value.

Although GWAS studies in cassava have made progress, research on high protein content remains in its infancy. In other crops, GWAS has been successfully applied to identify loci controlling important traits. For example, Chen et al. [8] identified 36 loci associated with 11 agronomic traits in cassava and detected several significant SNPs linked to stem thickness. Cheng et al. [9] performed GWAS on 147 cassava accessions and identified 155 markers significantly associated with latex yield. Huang et al. [10] used GWAS to uncover multiple loci related to disease resistance in cassava. These studies not only revealed the genetic basis of key cassava traits but also provided valuable gene resources for molecular breeding.

In model species, GWAS has also successfully identified genes related to seed protein content. Chen et al. [11] identified multiple SNPs significantly associated with protein content in maize (*Zea mays*). Li et al. [12] detected loci linked to protein content in soybean (*Glycine max*), laying a foundation for genetic improvement. Wang et al. [13] identified protein-related loci in rice (*Oryza sativa*) through GWAS. These findings elucidated the genetic architecture of protein accumulation in model species and provided critical resources for molecular breeding. Therefore, this study aimed to identify candidate genes underlying high storage root protein content in cassava through GWAS, providing a theoretical basis for breeding high-protein varieties.

In this study, we constructed a population of 261 cassava accessions and obtained high-density SNP markers through whole-genome resequencing [14]. To ensure data accuracy and reliability, we measured storage root protein content for three consecutive years using a multi-year, same-site phenotyping approach to minimize environmental effects [15]. Based on these data, we identified high-protein germplasm resources and ultimately selected 21 high-protein cassava lines. Deep genotypic analyses identified 8,171,448 SNPs associated with protein content, of which eight loci were significantly associated with the trait. We further shortlisted seven candidate genes with potential functional relevance. To validate the roles of these candidates in protein synthesis, we performed RT-qPCR to analyze their expression levels in high- and normal-protein lines. Six candidate genes were significantly up-regulated in high-protein genotypes. Collectively, these findings identify high-protein genotypes within the cross population, uncover key genes responsible for enhancing protein accumulation in cassava storage roots, and provide a theoretical foundation for breeding high-protein cassava lines and for understanding protein allocation mechanisms in this important crop.

## 2. Results

### 2.1. Statistical Analysis of Phenotypic Traits and Screening of High-Protein Varieties

During 2021 to 2023, the protein content in storage roots of 261 inbred lines from the cross (SC205 × 18R) was evaluated at the experimental field located in Haikou, Hainan Province, China

**Figure 1:**
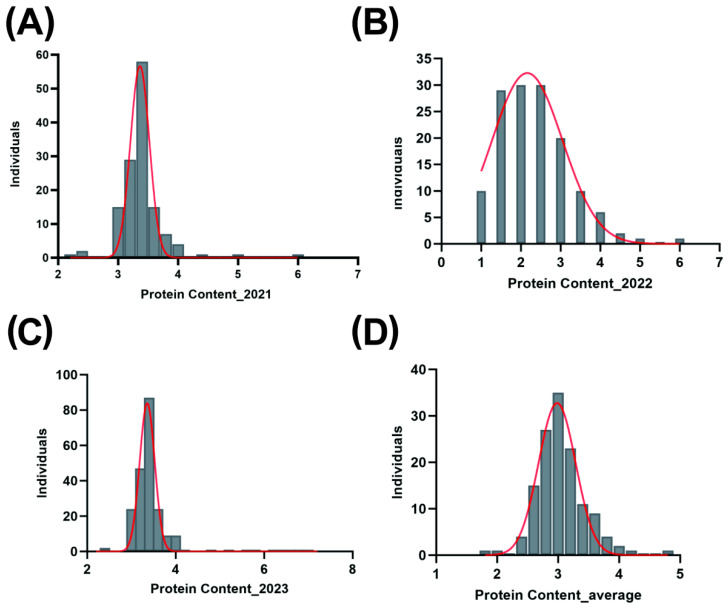
Frequency distribution of protein content phenotype. (**A**) Normal distribution of protein content in 2021. (**B**) Normal distribution of protein content in 2022. (**C**) Normal distribution of protein content in 2023. (**D**) Normal distribution of the average protein content from 2021 to 2023.

The statistical results of protein content are as follows: In 2021, the protein content ranged from 2.25% to 6.07%, with a mean of 3.40%, a standard deviation of 0.39, and a coefficient of variation (CV) of 11.60%. In 2022, the protein content ranged from 1.01% to 5.76%, with a mean of 2.37%, a standard deviation of 0.87, and a CV of 36.84%. The unusually high CV in 2022 may be attributed to environmental fluctuations, differences in soil fertility, or genotype × environment (G×E) interactions, which could have amplified phenotypic variation under specific field conditions. In 2023, the protein content ranged from 2.25% to 7.12%, with a mean of 3.42%, a standard deviation of 0.47, and a CV of 13.65%. The average protein content over the three years ranged from 1.87% to 4.87%, with a mean of 3.05%, a standard deviation of 0.40, and a CV of 13% (Table 1). Additionally, normality tests were conducted on the cassava protein content data in this study, and the results indicated that the distribution generally conforms to a normal distribution (Figure 1), thereby validating the rationality of the data for subsequent genome-wide association study (GWAS) analyses. The estimated broad-sense heritability (H^2^) for protein content was 0.68, indicating that genetic factors account for a substantial proportion of the observed phenotypic variation. The relatively high heritability suggests that protein content is a genetically stable trait across years. Furthermore, the inclusion of year as a random effect revealed limited environmental influence, supporting the reliability of selection based on this trait.

There are 21 lines exhibiting protein content exceeding 4% in the segregated population, and these lines can be used for comprehensive trait evaluation or serve as important resources for the breeding of new high-protein varieties (Table 2).

### 2.2. Distribution and Detection of Genomic Variation Sites

**Figure 2:**
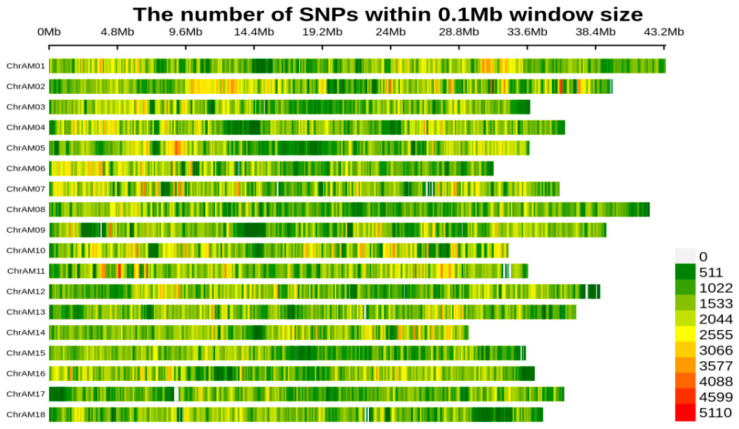
The distribution of SNP variant sites on 18 chromosomes. The number of SNPs is counted at 0.1 Mb intervals, with colors based on the count values.

Using SAMtools and VCFtools software to analyze the resequencing data of all 261 individuals in the F1 population, we identified all SNP and InDel variant sites. A total of 10,979,662 variant sites were detected, including 9,492,377 SNPs and 1,487,285 InDels. These variant sites are evenly distributed across the 18 chromosomes of cassava. We quantified the SNP frequency using 0.1 Mb bins and visualized the distribution through a heatmap of SNP density (Figure 2). Specifically, we observed that 52.56% of SNPs are located in intergenic regions, indicating that the majority of variations occur in these areas; nonsynonymous mutations account for 1.62%, suggesting that a small proportion of variations may lead to amino acid substitutions and thus affect protein function; 2.75% are located in exons, indicating potential functional impacts in coding regions; and 7.19% are in introns, suggesting possible roles in splicing regulation or gene expression regulation. These results provide essential foundational data for subsequent genome-wide association studies. Detailed statistical information on variant types, numbers, and proportions is presented in Table 3.

### 2.3. Genome-Wide Association Study of Storage Root Protein Content

**Figure 3:**
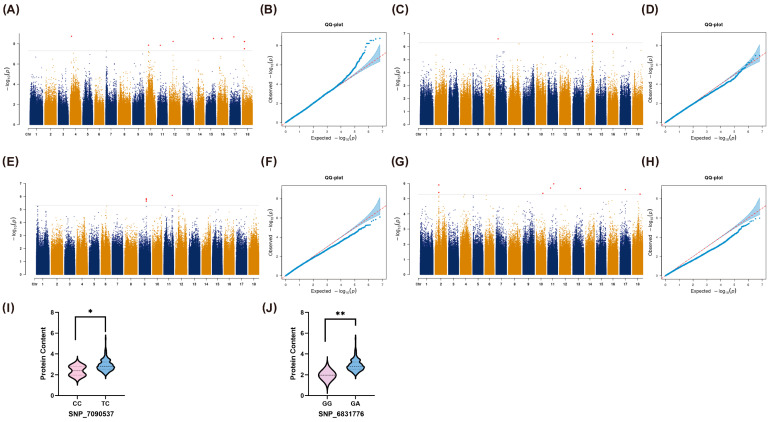
Manhattan and quantile–quantile (Q–Q) plots summarizing the GWAS results and haplotype analyses. The Manhattan plots illustrate the genome-wide distribution of SNP associations with storage root protein content across individual years and the three-year average. The x-axis represents chromosomal positions, and the y-axis shows –log_10_ (*p*-values), indicating the strength of association. The dashed horizontal line marks the significance threshold (*p* ≤ 1 × 10^−5^), and red dots highlight SNPs that surpass this threshold. The Q–Q plots assess the deviation between observed and expected *p*-values. The upward deviation from the diagonal line indicates the presence of true association signals with minimal inflation, confirming the robustness of the GWAS model. (**A**) Manhattan plot for 2021 storage root protein content. (**B**) Q–Q plot for 2021 storage root protein content. (**C**) Manhattan plot for 2022 storage root protein content. (**D**) Q–Q plot for 2022 storage root protein content. (**E**) Manhattan plot for 2023 storage root protein content. (**F**) Q–Q plot for 2023 storage root protein content. (**G**) Manhattan plot for the three-year (2021–2023) mean storage root protein content. (**H**) Q–Q plot for the three-year (2021–2023) mean storage root protein content. (**I**) Haplotype analysis of SNP_7090537 on chromosome 16. (**J**) Haplotype analysis of SNP_6831776 on chromosome 15. A, T, C, and G denote nucleotide bases; * indicates *p* < 0.05 and ** indicates *p* < 0.005.

In this study, a total of 8,171,488 quality-filtered SNPs that passed Hardy–Weinberg equilibrium were subjected to genome-wide association analysis against storage root protein content measured across the 2021–2023 seasons; Manhattan (Figure 3A–D) and quantile–quantile (Figure 3E–H) plots were generated for each dataset.

In 2021, GWAS of storage root protein content identified six significant SNPs distributed across six chromosomes, each contributing differentially to the trait: 0.16 for SNP_1641946 on Chr 4, 0.18 for SNP_4878878 on Chr 11, 0.17 for SNP_5378426 on Chr 12, 0.35 for SNP_6831776 on Chr 15, 0.35 for SNP_7090537 on Chr 16, and 0.19 for SNP_7485551 on Chr 17 (Table 4). Haplotype analyses of the two loci with the highest effects (0.35) revealed significantly higher protein levels in heterozygous genotypes than in homozygotes, indicating dominant or heterotic effects and suggesting that the ALT allele may regulate protein accumulation via incomplete dominance or recessive deleterious impact (Figure 3I,J).

In 2022, GWAS identified four significant SNPs on three chromosomes: 0.19 for SNP_2959024 on Chr 7, 0.17 and 0.20 for SNP_6427875 and SNP_6428019 on Chr 14, respectively, and 0.17 for SNP_7117572 on Chr 16 (Table 4).

In 2023, GWAS identified four significant SNPs on two chromosomes: 0.15, 0.16, and 0.15 for SNP_4104234, SNP_4104235, and SNP_4104236 on Chr 9, respectively, and 0.16 for SNP_5140201 on Chr 11 (Table 4).

Across the three-year mean, GWAS identified eight significant SNPs on six chromosomes: 0.15 for SNP_792191 on Chr 2, 0.12 for SNP_792192 on Chr 10, 0.13 and 0.14 for SNP_4622659 and SNP_4878249 on Chr 11, respectively, 0.14 for SNP_5017565 on Chr 13, 0.15 for SNP_5090879 on Chr 17, and 0.12 for SNP_7536143 on Chr 18 (Table 4).

Integrating GWAS results across three consecutive field seasons (2021–2023), we identified 21 significant SNPs associated with storage root protein content on 13 chromosomes. Specifically, these include SNP_792192 on Chr02 (2023), SNP_1641946 on Chr04 (2021), SNP_2959024 on Chr07 (2022), SNP_4104234, SNP_4104235 (2022) and SNP_4104236 (2023) on Chr09, SNP_4622659 on Chr10 (2023), SNP_4878878 (2021) and SNP_5140201 (2023) together with the three-year-mean SNPs SNP_4878249 and SNP_5017565 on Chr11, SNP_5378426 on Chr12 (2021), the three-year-mean SNP_5090879 on Chr13, SNP_6427875 and SNP_6428019 on Chr14 (2022), SNP_6831776 on Chr15 (2021), SNP_7090537 (2021) and SNP_7117572 (2022) on Chr16, SNP_7485551 (2021) and the three-year-mean SNP_7536143 on Chr17, and the three-year-mean SNP_8079404 on Chr18. Notably, SNP_6831776 (Chr15) and SNP_7090537 (Chr16) exhibited the largest effects in 2021 (R^2^ = 0.35 each), and their heterozygous genotypes consistently conferred superior protein content in subsequent haplotype analyses. Additionally, SNP_7536143 (Chr17) remained stably associated across the three-year mean dataset, indicating robust year-to-year consistency. Collectively, these loci provide reliable targets for marker-assisted breeding of high-protein cassava.

### 2.4. Identification of Candidate Genes

To validate the rationale for defining candidate intervals, we first performed linkage disequilibrium (LD) decay analysis. The results showed that r^2^ values remained above 0.30 within a 200 kb range, indicating strong LD in this region and supporting the use of ±100 kb windows for candidate gene identification around significant SNPs. The LD decay trend is illustrated in Supplementary Figure S1, and average r^2^ values at key distances (50, 100, 150, 200, and 250 kb) are summarized in Supplementary Table S1.

**Figure 4:**
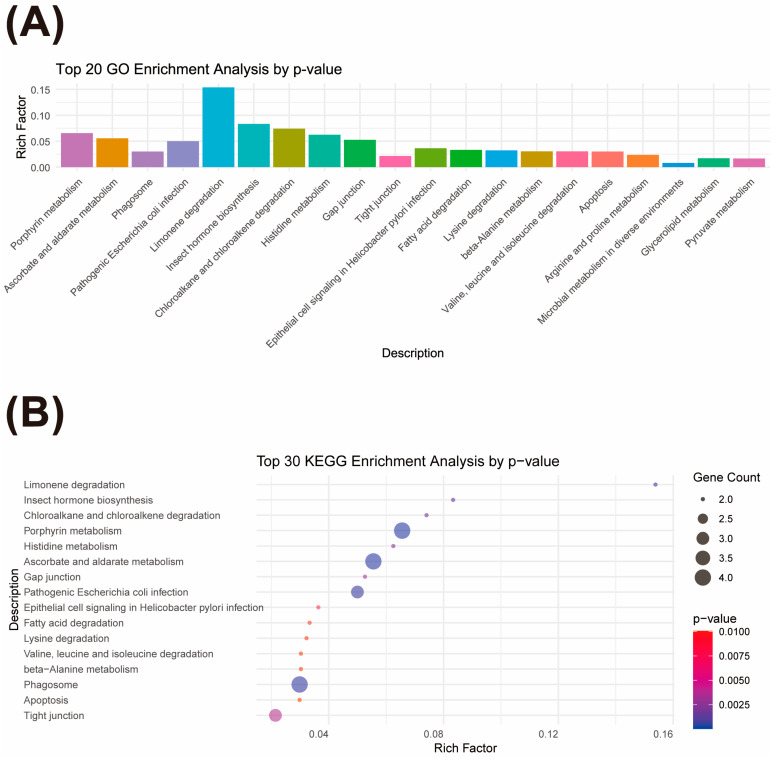
GO and KEGG enrichment analyses of candidate genes. (**A**) Bar plot of GO enrichment analysis for GWAS candidate genes, showing the top 20 GO terms with the smallest FDR values (most significantly enriched). (**B**) Bubble plot of KEGG enrichment analysis for GWAS candidate genes, showing the top 30 pathways with the smallest *p* values (most significantly enriched).

Subsequently, based on the analysis using the snpEff5.0e software, candidate genes were screened according to the specific genomic regions where high-quality SNPs were located (e.g., intergenic regions, upstream or downstream regions of genes). The genomic regions spanning 100 kb around each significant SNP were precisely captured and compared with the genomic annotation files, ultimately yielding 82 candidate genes. In this study, detailed Gene Ontology (GO) and Kyoto Encyclopedia of Genes and Genomes (KEGG) enrichment analyses were conducted on these 82 candidate genes to elucidate their potential roles in biological functions and metabolic pathways. The results of GO enrichment analysis indicated that these candidate genes are significantly involved in a variety of biological processes, including but not limited to amino acid metabolism, carbohydrate metabolism, energy metabolism, cellular structural composition, and responses to environmental stimuli. These processes are crucial for the regulation of protein content in plant storage roots, suggesting that these candidate genes may play a key role in regulating the synthesis and accumulation of storage root proteins (Figure 4A). KEGG enrichment analysis further clarified the specific metabolic pathways in which these candidate genes are involved, such as photosynthesis, starch and sucrose metabolism, glycolysis, and the circadian rhythm regulation associated with flowering. These enrichment results emphasize the potential roles of the candidate genes in energy conversion, material metabolism, and growth and development, particularly in regulating storage root protein content (Figure 4B).

### 2.5. Quantitative Analysis of Candidate Genes

**Figure 5:**
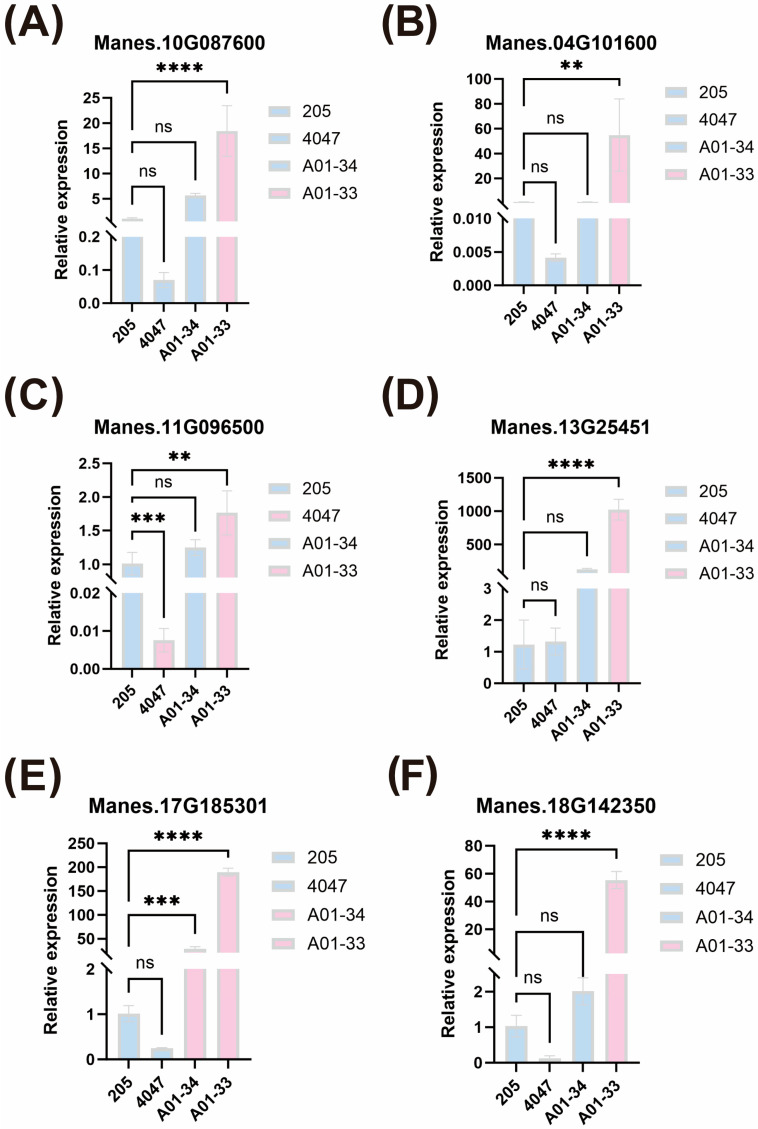
Expression of candidate genes in high-protein varieties, normal-protein varieties, and Wild species. Error bars represent the mean variance of three biological replicates. ** indicates *p* < 0.01, *** indicates *p* < 0.001, **** indicates *p* < 0.0001, and ns indicates non-significant.

To verify the accuracy of gene expression in cassava varieties with different protein content, Reverse Transcription-quantitative PCR (RT-qPCR) was performed to profile the transcriptional abundance of the seven candidate genes exhibiting higher *p*-values, using storage root tissues from the present population: two high-protein lines (A01-33 and A01-34), one conventional-protein cultivar (SC205), and the wild accession (4047). The results showed that, compared to the normal-protein variety (SC205), six candidate genes (*Manes.10G087600*, *Manes.04G101600*, *Manes.11G096500*, *Manes.13G25451*, *Manes.17G185301*, and *Manes.18G142350*) were significantly upregulated in the high-protein line (A01-33) (Figure 5). Among them, the gene *Manes.17G185301* was significantly upregulated in both high-protein lines (A01-33 and A01-34) (Figure 5E). This indicates that the results of our genome-wide association study are reliable.

Additionally, the candidate gene *Manes.04G101600*, located on chromosome 11, was identified as a member of the heat shock protein 40 family, DnaJ 8 (Table 5). DnaJ 8 belongs to the Hsp40 family and has been reported to be involved in folding regulation under high protein load conditions in animals and microorganisms [16]. Further RT-qPCR validation revealed that this gene is highly expressed in the storage root tissues of high-protein cassava varieties. The molecular chaperone protein encoded by this gene may support the high protein synthesis demands of these tissues by maintaining protein homeostasis.

## 3. Discussion

Cassava, a globally important food crop, is rich in starch in its storage roots, but the protein content of its storage roots has long been at a low level, far below that of major crops such as wheat, rice, and soybeans. This greatly limits its potential as a high-nutrient staple food [17,18]. Therefore, increasing the protein content of cassava storage roots is of great significance for enhancing its nutritional and economic value and can also enhance its potential for application in feed, industrial starch, and energy sectors [4]. However, research on candidate genes for cassava storage root protein content is currently in its infancy. This study, based on genome-wide association studies (GWAS), is the first to explore candidate genes related to cassava storage root protein content, which will help promote cassava precision molecular breeding and accelerate the selection of high-protein, high-yielding, and high-quality varieties. Compared with other major crops, genome-wide association studies (GWAS) targeting protein-related traits have made significant progress in species such as maize and soybean. For example, Zhang et al. identified multiple QTLs associated with seed protein content in soybean and found that the candidate genes were involved in amino acid metabolism and transport [19]. Similarly, Li et al. mapped SNPs linked to kernel protein accumulation in maize and validated their roles in nitrogen metabolism pathways [20]. These studies demonstrate that protein content is regulated by complex genetic mechanisms and is closely related to nutrient synthesis. In contrast, the genetic basis of protein traits in cassava remains largely unexplored. This study fills that gap by systematically identifying stable SNPs associated with protein content in cassava for the first time. Through expression validation, we further screened candidate genes with potential biological functions, providing new molecular targets for nutritional improvement in root crops.

The phenotypic analysis in this study revealed significant differences in cassava storage root protein content among years and varieties. Although environmental factors have a considerable impact on protein content (e.g., the coefficient of variation reached 36.84% in 2022), through the integrated analysis of three years of data, we successfully screened out 21 high-protein varieties with stable protein content above 4%. This result confirms the potential for transgressive segregation of storage root protein content in cassava germplasm, laying the foundation for subsequent genetic analysis. Notably, the normal distribution of protein content (Figure 1) indicates that this trait is controlled by multiple genes, consistent with the characteristics of quantitative traits, which also explains why the GWAS method is suitable for the study of such traits.

GWAS identifies associations between genotypes and phenotypes by testing hundreds of thousands of genetic variations in individuals with ancestral similarities but phenotypic differences. With the advancement of technologies such as next-generation sequencing (NGS), a large number of single-nucleotide polymorphism (SNP) markers have been identified, leading to a significant increase in marker density [21]. In recent years, the application of GWAS in cassava research has gradually increased, and genes related to important traits such as yield, disease resistance, and starch content in cassava have been identified [22]. This study represents a pioneering effort to use GWAS to identify candidate genes related to storage root protein content. In this study, high-quality SNP sites were used to lay the foundation for comprehensive genome-wide association studies for further research. Based on previous studies, to reduce the impact of environmental factors on protein content measurement, this study adopted a method of measuring at the same location for three consecutive years and combined statistical analysis to ensure the reliability of phenotypic data for subsequent association analysis. High-density SNPs were obtained by resequencing the whole genome at an average depth of 20×. Finally, after quality control and filtering, 8,171,448 high-quality SNP sites were obtained.

In this study, candidate genes were delineated by functionally annotating SNPs significantly associated with storage root protein content. Across GWAS runs for each yearly dataset and for the three-year mean, 21 loci exceeded the significance threshold; among these, SNP_6831776 on chromosome 15 and SNP_7090537 on chromosome 16 each explained 35% of the phenotypic variance, representing the largest genetic effects detected. Haplotype analyses of these loci further demonstrated that heterozygous mutant genotypes are strongly linked to elevated protein levels, indicating pronounced heterozygous advantage. These allelic configurations are therefore likely to play pivotal roles in protein synthesis or accumulation, potentially reflecting functional divergence during protein biosynthesis or intracellular trafficking.

GWAS association analysis identified 82 candidate genes. The screening of these candidate genes was based on SNPs associated with the protein content trait. Further functional annotation analysis indicated that these genes may be involved in key biological processes such as amino acid metabolism, carbohydrate metabolism, and energy metabolism, which are highly consistent with the biological demands of high-protein synthesis in storage roots. Of particular interest is the heat shock protein 40 family member *Manes.04G101600* (DnaJ 8), which has been proven to be involved in protein homeostasis maintenance in animals and microorganisms.

Recently, the combination of GWAS and RT-qPCR has been used to explore candidate genes. GWAS or RT-qPCR has been widely used to identify genes related to waterlogging tolerance, disease, growth, and development [23]. For example, in maize, Li et al. identified SNPs associated with alkali stress tolerance through GWAS and screened out five differentially expressed genes as key candidate genes through RT-qPCR analysis [24]. In another study, Xue et al. conducted GWAS on 277 accessions of mung bean, located 19 QTL regions, and screened out six hub genes through RT-qPCR analysis. These genes were significantly expressed in alkali-tolerant materials, indicating potential functions in alkali stress response [25]. In this study, RT-qPCR analysis was performed on seven candidate genes located at the eight significant loci identified through GWAS association analysis. It was found that six of the genes were significantly upregulated in high-protein varieties, among which *Manes.17G185301* was significantly highly expressed in both high-protein varieties. It is speculated that they exhibit specific expression patterns in biological processes such as protein synthesis, amino acid metabolism, and nutrient transport, further supporting their potential functions in protein content regulation. These findings not only promote the improvement of cassava nutritional quality but also provide new ideas for protein metabolism research in other tuber crops.

## 4. Materials and Methods

### 4.1. Plant Materials and Field Trials

The experimental population used in this study was cultivated in Haikou County, Hainan Province (110.00° E, 19.75° N). The population originated from the progeny of a cross between 18R and SC205. Among the 261 progeny individuals, some exhibited significantly higher protein content than their parents, demonstrating a phenomenon of heterosis. Therefore, this population was selected as the research subject for whole-genome resequencing and phenotypic analysis. The planting site was chosen in a flat area with fertile soil, and a randomized block design was employed for arrangement. The planting density was set at a row spacing of 1.0 m and a plant spacing of 0.8 m, with six plants per row [15].

Broad-sense heritability (H^2^) for protein content was estimated using a linear mixed-effects model implemented in the lme4 package in R. The model included genotype (Line) and year (Year) as random effects. Variance components were extracted to calculate H^2^ using the following formula: H^2^ = σ^2^_G/[σ^2^_G + (σ^2^_E/L × R)] is the residual variance, LL is the number of years, and RR is the average number of replicates per genotype. During the planting process, efforts were made to ensure complete seedling establishment in a single sowing, with a sufficient amount of basal fertilizer applied at once. A total of 40 kg of compound fertilizer containing equal amounts of nitrogen, phosphorus, and potassium (15-15-15) was applied per acre. During the growth period of cassava seedlings, two tillage operations were conducted to maintain soil looseness and reduce weed competition. When the cassava seedlings reached a height of 30 cm, thinning was performed to retain only one plant per hole, ensuring adequate space for plant growth. Appropriate measures were also taken to prevent common diseases and pests, such as bacterial leaf spot and red spider mites. When the cassava seedlings grew to a height of 60 cm, hilling-up was conducted to enhance the plants’ lodging resistance and prevent waterlogging. The growth cycle of cassava is approximately 10 months; after this point, the plants were harvested. Genotyping was performed on the 261 cassava materials, and protein content analysis was conducted from 2021 to 2023.

### 4.2. Phenotypic Trait Measurement

During the period from 2021 to 2023, precise measurements of protein content were conducted on 261 cassava samples. In the experiment, 0.2 g of cassava sample was accurately weighed and placed into a 100 mL digestion tube, ensuring that the sample did not adhere to the neck of the tube to minimize error. Five milliliters of concentrated sulfuric acid was added and allowed to stand for several hours to promote digestion. Subsequently, hydrogen peroxide was added in portions, with a bent-neck funnel placed over the tube, and the mixture was processed in a digestion furnace at 380 °C for 1 h. After cooling, an additional 1 mL of hydrogen peroxide was added, and this process was repeated 2–3 times until the solution became clear and transparent. The mixture was then heated for an additional 5–10 min to remove residual hydrogen peroxide. After digestion, the sample was cooled, the funnel was rinsed, and the rinsing solution was transferred into the digestion tube. The solution was then transferred to a 50 mL volumetric flask, mixed thoroughly, and set aside for later use. Three blank controls were also prepared to ensure the accuracy of the measurements [26,27].

Prior to distillation, sodium hydroxide solution, standard sulfuric acid solution, and mixed indicator were prepared. The Kjeldahl apparatus was preheated and subjected to a blank distillation to clean the tubing and stabilize the readings. A volume of 5.00 mL of the sample solution was drawn into the distillation tube for measurement, and the data were recorded.

Phenotypic statistical analysis of protein content was performed based on measurement data collected consecutively from 2021 to 2023. First, varieties with protein content reaching 4% or higher across the three-year measurements were identified and classified as high-protein cassava varieties. The three-year average protein content was then calculated for each sample to reflect its long-term stable protein level. Further statistical parameters, including maximum (Max), minimum (Min), standard deviation (SD), and coefficient of variation (CV), were computed to comprehensively evaluate the variability in protein content. Additionally, phenotypic data histograms were generated using the hist function in R 4.4.0 software to visually display the distribution characteristics of protein content across different years, facilitating assessment of the distribution patterns.

### 4.3. Genotyping and Polymorphism Analysis

In this study, genomic DNA was extracted from fresh leaves (5–8 g) of different cassava materials using an improved CTAB method. Subsequently, ASFM libraries were prepared using two restriction enzymes, and bacterial liquid PCR amplification was performed after library construction. The bacterial liquid PCR products were sent for sequencing. If the sequencing results contained the adapter sequences designed for ASFM technology, the corresponding PCR products were used to construct small-fragment sequencing libraries, which were then sequenced on the Illumina Hi-Seq 2500 platform [8]. Leaf samples from each cassava accession were collected for sequencing, with a sequencing depth of 20×, yielding 15 G of raw data per sample. The raw Illumina sequencing data were subjected to quality control and data filtering using Perl scripts, and the total number of reads obtained during sequencing was counted. Based on the barcodes designed for ASFM technology, samples were classified, and the number of effective reads in each sample from different cassava materials was calculated [9,28].

The quality-optimized sequencing reads were aligned to the cassava AM560 reference genome using the Bowtie 2 v2.5.1 software, and SNP and InDel variant sites were detected using SAMtools and VCFtools [29]. To ensure data quality, filtering was performed using VCFtools based on the following criteria: Hardy–Weinberg Equilibrium (HWE) > 0.001 and Minor Allele Frequency (MAF) ≥ 0.05. Low-quality variant sites were discarded to obtain high-quality variant sites [30]. Additionally, the following parameters were used to evaluate SNPs and InDels: “QD < 2.0, FS > 200.0, SOR > 10.0, MQRankSum < −12.5, ReadPosRankSum < −8.0”. Finally, the snpEff software was used for annotation, categorizing the positions of SNPs into intergenic regions, untranslated regions (including 3′ and 5′ untranslated regions), intronic regions, and coding regions [31,32].

### 4.4. Genome-Wide Association Study

GWAS is a genetic research method aimed at identifying genetic variants, typically SNPs, associated with specific traits or diseases. In this study, we conducted a GWAS on cassava protein content using high-quality SNP sites that had undergone rigorous filtering. The association analysis was performed using the mixed linear model (MLM) in the EMMAX (emmax-beta-07Mar2010) software [8,33]. Prior to the analysis, Plink v1.90b6.12 software was used to adjust the data format, converting the VCF file to the tfam and tped formats recognizable by EMMAX. The kinship matrix generated by EMMAX was utilized to correct for population structure and random polygenic effects. A significance threshold of *p* ≤ 1 × 10^−5^ was set to identify significant GWAS signals associated with cassava protein content. This threshold was set as 0.05/n, where n represents the total number of SNP markers tested, to control for multiple testing and reduce false positives.

LD decay analysis was performed using PopLDdecay on quality-filtered SNP data. The maximum physical distance was set to 1000 kb, with a binning scheme of 50 kb per interval. Only high-quality SNPs were included in the analysis, filtered based on missing rate (≤0.2), minor allele frequency (MAF ≥ 0.05), and Hardy–Weinberg equilibrium (HWE > 0.001). For each bin, the average pairwise r^2^ value was calculated to evaluate the decay of linkage disequilibrium with increasing physical distance. The Manhattan and QQ plots for visualizing the GWAS results were generated using the qqplot package in R.

### 4.5. Candidate Gene Identification and Functional Annotation

After completing the genome-wide association study (GWAS), we identified 8,171,488 SNPs significantly associated with protein content in cassava. Functional annotation analysis was subsequently conducted on these SNPs. Based on the annotation results obtained using the snpEff software, we screened for potential candidate genes according to the specific locations of the high-quality SNPs (e.g., intergenic regions, upstream/downstream of genes). SNPs located within genes were directly considered as candidate genes. If an SNP was situated in the upstream or downstream regions of a gene (e.g., promoter or terminator regions), the corresponding gene was also selected as a candidate. For SNPs located in intergenic regions, the nearest upstream or downstream genes, based on genetic distance, were designated as candidate genes. Utilizing the “bedtools closest” function from the Bedtools toolkit [34], we successfully extracted the candidate genes associated with these significant SNPs. Finally, we performed Gene Ontology (GO) and Kyoto Encyclopedia of Genes and Genomes (KEGG) enrichment analyses of candidate genes using the clusterProfiler package in R [35].

### 4.6. Candidate Gene Expression Analysis

To verify the expression of the genes identified in cassava varieties with different protein contents, we selected *actqf2* as the reference gene based on extensive literature comparison. We then performed RT-qPCR analysis on the seven candidate genes in high-protein varieties (A01-33 and A01-34), normal-protein variety (SC205), and the wild species (4047) within our population.

First, we collected tuber tissues from high-protein cassava varieties (A01-33 and A01-34), a normal-protein variety (SC205), and the wild species (4047). Total RNA was extracted using the TRIzol method, and RNA purity and concentration were measured using a NanoDrop-2000 spectrophotometer. Subsequently, RNA was reverse-transcribed into cDNA using the HiScript III RT SuperMix for qPCR to ensure the accuracy of the subsequent RT-qPCR reactions [36]. Primers were designed based on the target gene sequences using Primer3plus, with *actqf2* serving as the reference gene [37]. RT-qPCR was performed using SYBR Green PCR Master Mix for fluorescence detection, with a reaction volume of 20 µL and run on an ABI 7500 Fast Real-Time PCR System. All samples were analyzed in triplicate, and the relative expression levels of the key candidate genes were analyzed by calculating the 2^−ΔΔCt^ values [38]. Finally, we compared the gene expression levels between the high-protein cassava group and the wild species and normal-protein variety group, and assessed their regulatory roles in high-protein cassava.

## 5. Conclusions

Through the continuous three-year measurement and evaluation of the protein content in the storage roots of 261 lines of the hybrid experimental population SC205*18R, it was found that there were 21 lines with high protein content that was stably above 4%. A total of 22 significant associated loci of protein content in storage roots were identified through genome-wide association analysis, with their contribution rates ranging from 0.12 to 0.35. For instance, the haplotypes of SNP-6831776 and SNP-7090537 have a prominent contribution to the protein content in the storage roots and can be used as major-effect markers in breeding. Meanwhile, using quantitative RT-qPCR, it was found that 6 of 82 candidate genes were markedly up-regulated in high-protein varieties, with Manes. 17G185301 showing consistently superior expression. These results provide critical molecular markers and gene resources for marker-assisted breeding of high-protein cassava and deepen our understanding of protein allocation in tuber crops.

## Figures and Tables

**Table 1:** Protein content variation in storage root of cassava.

Trait	Year	Range	Mean	SD	CV
Protein Content	2021	2.25–6.07%	3.40%	0.39	0.12
	2022	1.01–5.76%	2.37%	0.87	0.37
	2023	2.25–7.12%	3.42%	0.47	0.14

**Table 2:** Selected lines with higher protein in storage roots in the hybrid population.

Individuals	Protein Content	Individuals	Protein Content
A01-007	6.07%	A01-422	4.03%
A01-008	4.96%	A01-430	4.57%
A01-033	5.07%	A01-453	4.15%
A01-034	5.03%	A01-455	5.47%
A01-340	4.20%	A01-460	4.66%
A01-387	5.05%	A01-505	4.39%
A01-415	7.12%	A01-531	4.20%
A01-416	4.00%	A01-562	4.16%
A01-418	4.05%	A01-609	4.51%
A01-420	4.00%	A01-620	5.76%
A01-421	4.03%		

**Table 3:** Annotation statistics of SNP information. SNPs were categorized by genomic location and functional impact. The bottom three rows summarize overall genome metrics derived from the same annotation output.

Type	Count	Ratio
Downstream	2,666,688	17.83%
Exon	410,429	2.75%
Intergenic	7,858,808	52.56%
Intron	1,074,878	7.19%
Splice site acceptor	2331	0.02%
Splice site donor	2427	0.02%
Splice site region	27,842	0.12%
Upstream	2,780,654	18.60%
Missense variant	242,359	1.62%
Genome total length	645,399,631	
Genome effective length	645,399,631	
Total SNPs	9,492,337	

**Table 4:** Annotation information of significant loci.

Chromosome	Year	SNP	Position	*p*-Value	R^2^	REF	ALT
02	2023	SNP_792192	13148897	1.23 × 10^−6^	0.15	C	T
04	2021	SNP_1641946	4696409	1.80 × 10^−9^	0.16	G	A
07	2022	SNP_2959024	7028219	2.54 × 10^−7^	0.19	A	C
09	2022	SNP_4104234	24914148	1.77 × 10^−6^	0.15	T	C
		SNP_4104235	24914151	1.49 × 10^−6^	0.16	C	G
	2023	SNP_4104236	24914164	2.28 × 10^−6^	0.15	A	G
10	2023	SNP_4622659	22538541	4.41 × 10^−6^	0.12	A	C
11	2021	SNP_4878878	9927394	1.44 × 10^−8^	0.18	G	A
	2023	SNP_5140201	28175312	8.09 × 10^−7^	0.16	A	G
	2021–2023 Average	SNP_4878249	9889485	1.97 × 10^−6^	0.13	T	C
		SNP_5017565	20325110	1.05 × 10^−6^	0.14	G	A
12	2021	SNP_5378426	13017554	5.96 × 10^−9^	0.17	G	A
13	2021–2023 Average	SNP_5090879	25501246	2.13 × 10^−6^	0.14	C	A
14	2022	SNP_6427875	22931608	3.99 × 10^−7^	0.17	T	G
		SNP_6428019	22940512	1.08 × 10^−7^	0.20	T	C
15	2021	SNP_6831776	25558507	2.97 × 10^−9^	0.35	G	A
16	2021	SNP_7090537	13958220	2.97 × 10^−9^	0.35	T	C
	2022	SNP_7117572	16338879	1.12 × 10^−7^	0.17	T	C
17	2021	SNP_7485551	13939320	2.07 × 10^−9^	0.19	C	T
	2021–2023 Average	SNP_7536143	18444849	2.53 × 10^−6^	0.15	C	T
18	2021–2023 Average	SNP_8079404	25680373	4.94 × 10^−6^	0.12	G	A

**Table 5:** Annotation of candidate genes for protein content trait localization.

Chr	Pos	Gene	Description
02	13148897	Manes.02G165100	Belongs to the peroxidase family
10	22538541	Manes.10G087600	TIR domain
11	9889485	Manes.04G101600	Chaperone protein dnaJ 8
11	20325110	Manes.11G096500	N-acetyltransferase-like
13	25501246	Manes.13G254510	-
17	18444849	Manes.17G185301	-
18	25680373	Manes.18G142350	-

## Data Availability

Data for this study can be made available with a reasonable request to the authors. The data are not publicly available due to The data presented in this study are not publicly available due to privacy and ethical restrictions.

## Supplementary Materials

The following supporting information can be downloaded at: https://www.mdpi.com/article/10.3390/plants14203162/s1, Table S1. Average r^2^ values at key physical distances in the cassava population. Figure S1. LD decay curve showing the decline of linkage disequilibrium (r^2^) within a 200 kb range.
