# Supplementary material for: Genomic-Wide Association Markers and Candidate Genes for the High-Protein Trait in Storage Roots of Cassava (Manihot esculenta)
Source: Plants (Basel). 2025 Oct 15;14(20):3162. doi: 10.3390/plants14203162 (PMC12566772; doi:10.3390/plants14203162)
Supplement: Supplementary file 1 [file plants-14-03162-s001.zip › plants-3825755-supplementary.pdf]

Table S1. Average r^2 values at key physical distances in the cassava population.

Distance/kb	R^2
**50**	**0.39**
**100**	**0.37**
**150**	**0.37**
**200**	**0.37**
**250**	**0.36**

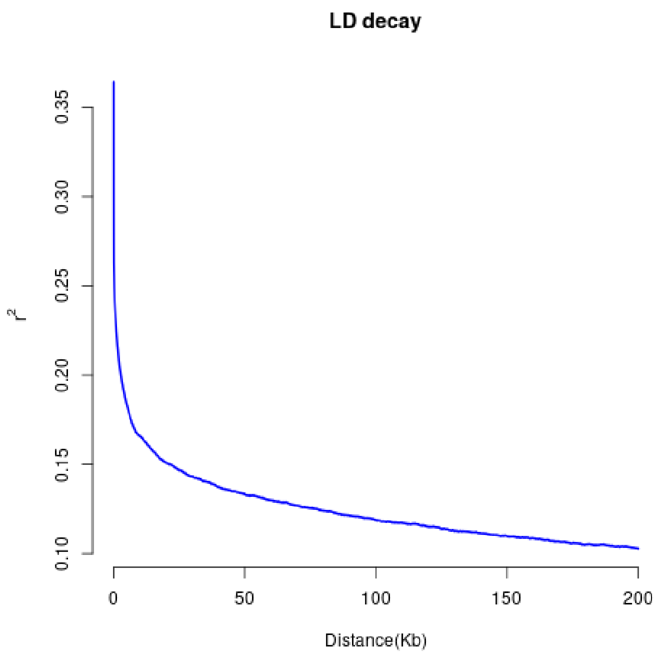

Figure S2. LD decay curve showing the decline of linkage disequilibrium (r^2) with
